# Multi-predator feeding aggregations: understanding dynamic communities through functional niches and demographic analysis tools

**DOI:** 10.1098/rstb.2025.0198

**Published:** 2026-10-15

**Authors:** Wednesday Davis, Rochelle Constantine, Gaia Dell'Ariccia

**Affiliations:** ^1^ Institute of Marine Science, The University of Auckland, Auckland, New Zealand; ^2^ School of Biological Sciences, The University of Auckland, Auckland, New Zealand; ^3^ Environmental Evaluation and Monitoring Unit, Auckland Council, Auckland, New Zealand

**Keywords:** foraging, community ecology, functional niche, marine, seabirds, cetaceans, New Zealand, Hauraki Gulf

## Abstract

Multi-predator feeding aggregations (MPFAs) are ocean events whereby two or more species forage on the same or co-occurring prey. Such communities are dynamic, with community structure reflecting the connections between species and the benefits and costs of associations. The analysis of MPFAs provides an overview of ecosystem health, with social networks reflecting prey and available resources. Demographic analyses of MPFAs can act as process-based indicators of community responses to rapid change. To understand complex community dynamics, we identified community types in MPFAs using hierarchical clustering and determined the social associations and roles of the different species present. We collected data on 179 MPFA events, recording 55.4 hours of behavioural observations from 33 843 individuals belonging to 19 seabird and three cetacean species in the Hauraki Gulf, New Zealand. Eight MPFA community types were identified, representing planktivorous, scavenger or polytrophic aggregations. Seabird–cetacean and seabird–seabird associations dominated species associations. We interpret the species’ roles by combining our observations of behavioural states with knowledge from the literature. The study of marine MPFAs offers a community-level perspective for understanding community dynamics and food-web organization under future ecosystem changes. Using social network analyses, we advance the understanding of species interconnectedness to better inform ecosystem-based conservation management.

This article is part of the theme issue ‘Multi-predator feeding aggregations in pelagic waters’.

## 1. Introduction

Multi-predator feeding aggregations (MPFAs) are dynamic ocean events whereby two or more species forage together on the same ephemeral prey patch. When predators detect underwater prey, they may herd it to the surface, acting as foraging facilitators, increasing prey availability for surface-feeding predators [1–3]. This activity may attract other predators using acoustic, visual and/or chemosensory signals [4], leading to the formation of an MPFA. While foraging near others can increase competition, using the behaviour of other predators to enhance foraging opportunities may outweigh these costs. Social contexts and predator–prey relationships are important structuring forces determining MPFA composition and species co-occurrence patterns, with social networks reflecting the processes that drive species to coexist [5].

Flexible foraging strategies and diverse foraging behaviours drive species to exhibit variable functional roles within MPFAs, with community composition reflecting the risk-reward trade-offs of foraging alongside con- and hetero-specifics at the sea surface [6]. Social networks reflect information sharing about food sources and predator presence. Individuals use the behaviour of others to locate resources and assess risk, influencing their own foraging decisions and leading to social structures that correlate with resource distribution. The structure of a social network, including who interacts with whom, reveals how this information spreads and can influence foraging efficiency and population dynamics [5]. MPFA persistence depends on the interactions between species, with roles terminating when the MPFAs are not beneficial or social structures degrade [7–9].

The demographic analysis of MPFAs provides an overview of ecosystem health, with social networks reflecting prey and available resources [5]. Marine megafauna, including seabirds, sharks and marine mammals, are often considered ocean sentinels, providing indications of anthropogenic stressors with the communities they form reflecting these across multiple trophic levels [10–12]. While species' aggregation around a prey source is well-recognized in community ecology studies [1,13], single-species studies of large marine animals are more predominant. These foraging relationships are essential for species' survival in an ever-changing ecosystem. However, the function and extent of these interactions vary between taxa, with interspecific interactions and facilitation influencing the organization of communities [14].

The study of marine MPFAs offers a community-level perspective for understanding community dynamics and food-web organization under future ecosystem change [6,15,16]. Demographic analyses of MPFAs can act as process-based indicators of community responses to rapidly changing ocean health. To better understand complex community dynamics of MPFAs, our research focused on (i) what community types can be found in MPFAs using hierarchical clustering, and (ii) what are the social associations and roles of different species engaged in MPFA events.

The Hauraki Gulf—Tīkapa Moana—Te Moananui-ā-Toi (hereafter, the Gulf) is well recognized for its biological diversity, productivity and the wide variety of marine ecosystems and marine predators it supports. However, with increasing environmental pressures, anecdotal evidence indicates the Gulf's MPFAs are reducing in size and number, with changes in prey availability driving shifts in prey selectivity [17] and association patterns [18]. These characteristics make the Gulf the perfect case study for testing and applying demographic analysis tools thereby revealing how MPFA dynamics can be used to facilitate a greater understanding of species roles and interconnectedness to better inform ecosystem-based conservation management.

## 2. Material and methods

This study was carried out in the Hauraki Gulf (36° 10′–37° 10′ S and 174° 40′–175° 30′ E) on the northeastern coast of Aotearoa New Zealand (figure 1). This productive ecosystem is a hotspot for marine megafauna including seabirds, sharks and cetaceans [19,20].

**Figure 1.**
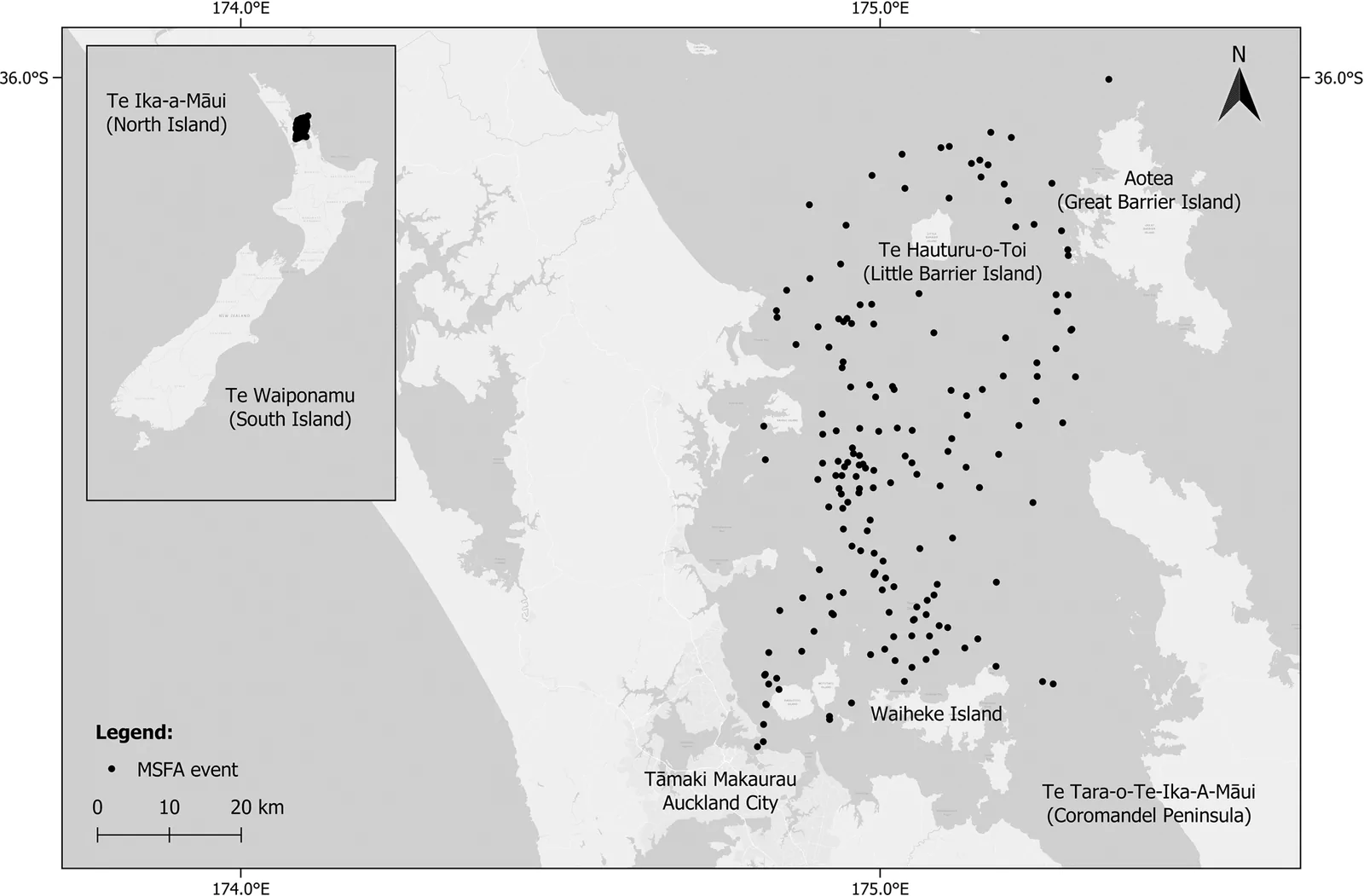
Map of the Hauraki Gulf study area (denoted by the black box on the inset map) with multi-predator feeding aggregation events shown by the black dots (*n* = 179).

Our study of MPFAs was conducted 2–3 days per month from September 2019 to August 2021 onboard the University of Auckland's 15 m research vessel, the RV *Hawere* and the 20 m whale watch vessel, the MV *Dolphin Explorer*. MPFA events were defined as aggregations of two or more species foraging in a coordinated manner [21]. Three to six observers used binoculars and the naked eye, employing continuous scanning methodology to detect signs of MPFA activity, including whale blow, surface disturbance and flying or diving seabirds. For each MPFA event, we recorded GPS location, and using continuous scan-sampling we recorded the species present, the number of individuals of each species, and their behaviours within the MPFA, with changes in the behavioural states of the different species recorded throughout the observation period. Recorded behaviours were classified as foraging, active non-foraging (e.g. flying), or non-active non-foraging (e.g. resting) (see the supplementary material, table S1 for classifications). MPFAs were observed until foraging activity stopped, time constraints forced the vessel to move on, or a representative sample of the event had been recorded, for example, repeated sequences of behaviours were observed by individuals of the same or different species during the event.

Analyses were conducted in RStudio (version 1.4.1717), PRIMER-e (version 7 + PERMANOVA) and SOCPROG (version 2.9). Within RS tudio, the *tidyr* package [22] was used for data management and reorganization and *ggplot* [23] and *igraph* [24] packages for data visualization.

### (a) Attendance

Attendance of species within MPFA events was determined as the percentage of workups each species and species group encountered, ranging between 0% = never attending and 100% = always attending. Mean group size of species was compared using a generalized linear mixed model (GLMM) with a quasi-Poisson error distribution. The effects of species (*n* = 22), cluster (*n* = 8), season (*n* = 4) and MPFA richness (number of species) on mean group size were tested. GLMM results were then investigated using a one-way analysis of variance (ANOVA) and pairwise comparison for factor levels. Owing to low attendance Caspian (*Hydroprogne caspia*) and other unidentified terns were all combined into ‘unidentified terns’. Likewise, owing to their functional and ecological similarity, white-faced (*Pelagodroma marina*), New Zealand (*Fregetta maoriana*) and other unidentified storm petrels were combined into ‘storm petrels’. While we primarily identified different shearwater and petrel species, there were occasions where this was not possible, so we combined these into ‘unidentified petrels/shearwaters’.

The Margalef species richness index [25] and Shannon diversity index [26] were calculated for each MPFA event to indicate the total number of species within an MPFA and species evenness and dominance within a community.

### (b) Cluster analysis

To investigate community composition, we generated a Bray–Curtis similarity index using PRIMER-e to determine variables influencing the observed community structure [27,28]. MPFA abundance data were square root transformed to reduce the dominant contribution of abundant species. Non-metric multidimensional scaling and hierarchical cluster analysis were performed using Primer-7 to determine community types (hereafter clusters). SIMPER (similarity pairwise comparison ANOSIM test) and SIMPROF test of multivariate structure within the dendrogram analysis were performed, clustering MPFAs by composition similarity and identifying species and their relative contribution to observed differences between MPFAs. MPFA composition was further tested using a non-parametric permutational multivariate analysis of variance (PERMANOVA), which analysis interactions between factors.

### (c) Social association index

Social association analysis was conducted in SOCPROG v2.9 using square root transformed data of the presence and absence of species in MPFAs [29]. Values for social association were calculated using the simple ratio method, using the co-occurrence of species to determine the degree of affinity (1 = always co-occurring and 0 = never) between species pairs. These indices were combined with the number of associating species (degrees) to generate a social association matrix and matrix plot using the package *igraph* [24]. Unidentified terns and unidentified petrels/shearwaters were excluded from this analysis. Because these groups comprise multiple species, their social connections would represent an aggregate of connections from different constituent species, some of which are already included in the model.

### (d) Behavioural states

Using the group focal follow data, we analysed the time that each species spent in each of the behavioural states (supplementary material, table S1). Using time stamps, sample intervals were indicators of the ‘duration’ of time the group spent in different behavioural states throughout the MPFA. To minimize the effects of group size, the proportion of the group performing the behaviours (from 0 = no individuals to 1 = all individuals) was used. The proportions were then used to summarize the group's engagement in the states foraging, active non-foraging and non-active non-foraging. Using the proportions for each sample interval, an overall sum of the proportion of time spent in each of the behavioural states throughout the MPFA duration was generated for each species group.

The proportion of time species spent in each behavioural state was analysed with a GLMM of the quasi-binomial error distribution for 0–1 proportion data, weighted by the mean group size of each species within MPFA events. Each explanatory categorical variable was investigated using a chi-squared ANOVA and pairwise comparisons with significance at *p* < 0.05. Overall activity budget GLMMs were carried out with the inclusion of all species. Only species observed in more than 5% of MPFAs were included (hereafter, ‘frequently occurring species’).

GLMM's testing the significance of the following variables: species, diversity, duration, richness and season were applied to data filtered by cluster and species to allow for investigation of within-cluster and within-species differences.

## 3. Results

During the two study years, we collected data on 179 MPFA events from 45 trips and 198 hours of survey effort. We conducted 20 trips on the university vessel and 24 trips on the whale-watch vessel, with an average survey duration of 5.2 and 3.4 hours, respectively, and a cumulative total of 55.4 hours of behavioural observations.

### (a) Attendance

We recorded a cumulative total of 33 843 individuals belonging to 22 categories: 16 seabird species, three cetacean species and three groupings of unidentified species (i.e. terns, petrels/shearwaters and storm petrels). Of these MPFA events, 33% (*n* = 59) only had seabirds and 67% (*n* = 120) involved both seabirds and cetaceans. MPFA event attendance (table 1) varied, with a mean of 3.7 ± 0.1 s.e. species and 158.6 ± 17.8 s.e. individuals attending, although MPFA size and richness were variable across events (range: 2–1936 individuals and 2–8 species, respectively). The most frequently attending species were the Australasian gannet (*Morus serrator*, *n* = 120), common dolphin (*Delphinus delphis*, *n* = 98) and fluttering shearwater (*Puffinus gavia*, *n* = 111).

**Table 1.** Species names, encounters and group sizes during MPFA events (*n* = 179).

species name	common name	number of encounters in MPFAs/%	mean/median group size	group size range
*Ardenna bulleri*	Buller's shearwater	43/24%	37/10	1–500
*Ardenna carneipes*	flesh-footed shearwater	71/40%	18/10	1–130
*Ardenna grisea*	sooty shearwater	7/4%	4/3	2–10
*Balaenoptera edeni brydei*	Bryde's whale	43/24%	1/1	1–6
*Chroicocephalus novaehollandiae scopulinus*	red-billed gull	14/8%	4/2	1–20
*Delphinus delphis*	common dolphin	98/55%	70/37.5	4–500
*Eudyptula minor*	little penguin	7/4%	2/2	1–3
*Larus dominicanus*	black-backed gull	31/17%	3/1	1–15
*Macronectes halli*	giant petrel	4/2%	2/1.5	1–5
*Morus serrator*	Australasian gannet	120/67%	77/50	1–600
*Pachyptila turtur*	fairy prion	2/1%	25/25	10–40
*Pelecanoides urinatrix*	diving petrel	11/6%	6/3	1–20
*Phalacrocorax varius*	pied shag	7/4%	4/1	1–20
*Procellaria parkinsoni*	black petrel	4/2%	43/35	3–100
*Pterodroma cookii*	Cook's petrel	7/4%	6/5	1–20
*Pterodroma gouldi*	grey-faced petrel	6/3%	16/7.5	1–50
*Puffinus gavia*	fluttering shearwater	111/62%	97/32.5	1–2000
*Sterna striata*	white-fronted tern	44/25%	63/30	1–600
*Tursiops truncatus*	bottlenose dolphin	2/1%	8/8	6–10
-	storm petrels	25/14%	14/5	1–100
-	unid. shearwater/petrel	1/1%	5/5	5
-	unid. tern	8/5%	9/2.5	1–30

Group size varied by species, cluster, season, Shannon diversity and Margalef's richness index (GLMM, ANOVA, *p* < 0.005). Group size (estimate ± s.e.) decreased with diversity (−1.69 ± 0.21) and increased (0.97 ± 0.25) with richness (*p* < 0.005). Pairwise comparison revealed that group sizes were smaller in autumn than winter (estimate ± s.e.; −0.55 ± 0.18; *p* = 0.01) and spring than winter (estimate ± s.e.; −0.7 ± 0.18; *p* = 0.01).

### (b) Cluster analysis

Hierarchical clustering analysis (Bray–Curtis similarity index) was performed on presence–absence data from 179 MPFA events, identifying eight unique clusters with significantly (*p* = 0.05) distinct MPFA communities using SIMPROF analysis. At each successive step of clustering, the two clusters with the greatest similarity are linked together. The horizontal axis indicates separation and dissimilarity between MPFA composition and the vertical axis, indicating the level of similarity between events (figure 2). Clusters a, c, f and g represent aggregations dominated by planktivorous predators, for example, Bryde's whales, storm petrels and fluttering shearwaters. The occurrence of cluster b reflects scavenging opportunities, with the MPFAs dominated by scavenging seabirds (e.g. gulls). Clusters d, e and h are indicative of polytrophic aggregations owing to the cumulative contribution of piscivorous and planktivorous species including common dolphins, Australasian gannets and fluttering shearwaters. The attendance of individual species to each cluster and their behaviours are in the supplementary material, figures S1 and S2 respectively. Of these eight clusters (table 2), cluster h was the most frequent, accounting for 66% (*n* = 118) of the 179 MPFA events. Cluster e was observed on 15% (*n* = 27) of the events, and all others were observed on 10 or fewer occasions. Similarity pairwise comparison (SIMPER; table 2) identified 10 species accounting for differences between MPFAs.

**Figure 2.**
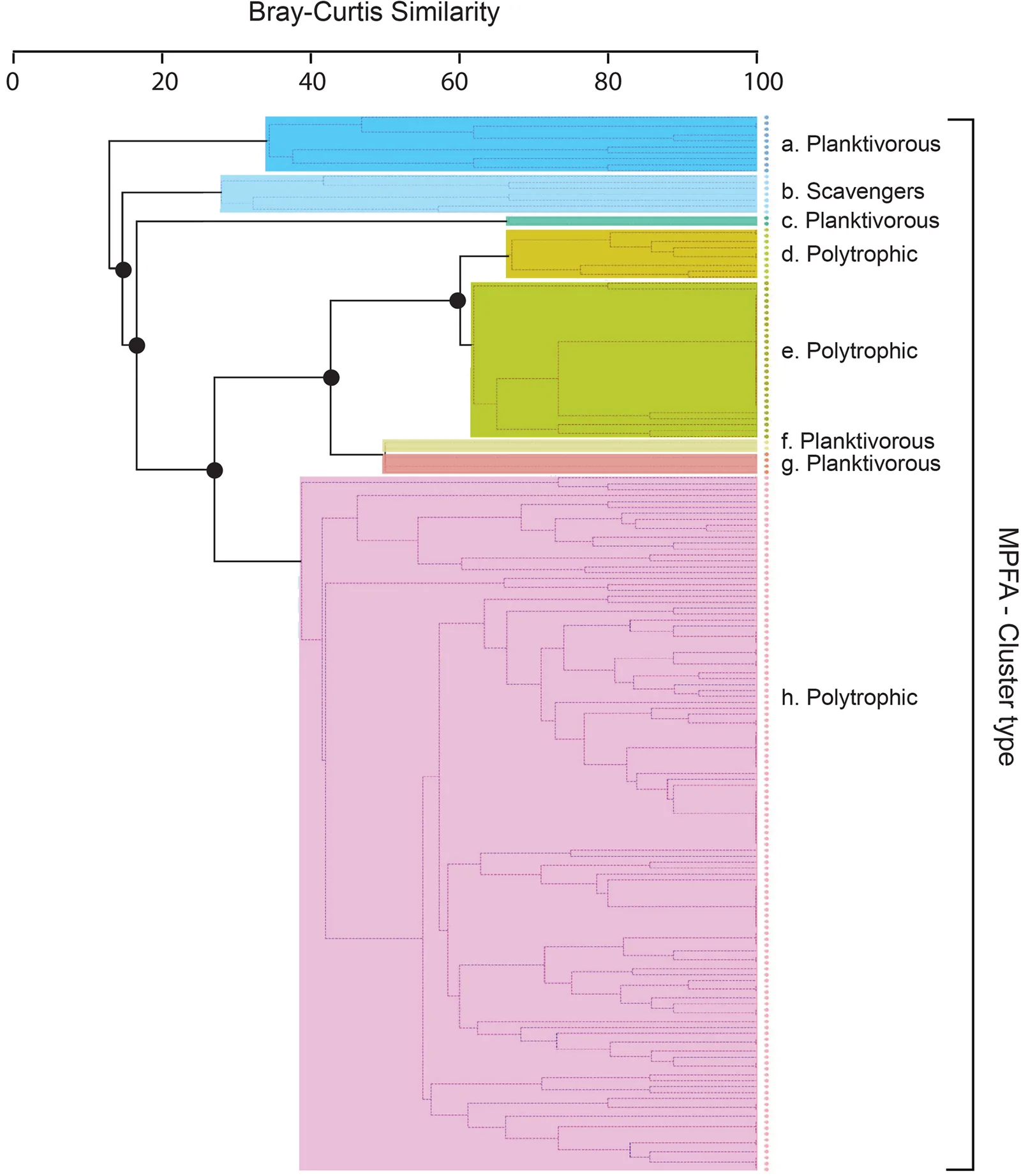
Hierarchical clustering dendrogram of the Bray–Curtis similarity of 179 presence–absence transformed MPFAs showing the eight clusters (a–h) accompanied by the predator composition descriptor (cluster type).

**Table 2.** Summary of the eight clusters with a similarity pairwise comparison analysis of the % contribution of each species to the similarity between MPFA events (‘*n* and %’) calculated from the Bray–Curtis similarity index (‘similarity’).

cluster	number of events/%	predator composition	similarity	main species	% contribution
a	10/5.6%	planktivorous	44.26	Bryde's whale	63.7
				storm petrel	16.6
b	7/4%	scavengers	35.1	black-backed gull	62.4
				red-billed gull	75.9
c	2/1%	planktivorous	66.67	Buller's shearwater	50
				fairy prion	50
d	9/5%	polytrophic	75.81	Australasian gannet	30.3
				fluttering shearwater	30.3
				white-fronted tern	30.3
e	27/15%	polytrophic	84.67	fluttering shearwater	49.5
				white-fronted tern	49.5
f	4/2%	planktivorous	50	fluttering shearwater	100
g	2/1%	planktivorous	58.33	fluttering shearwater	85.7
h	118/66%	polytrophic	55.44	Australasian gannet	38.9
				common dolphin	28.9
				fluttering shearwater	13.7

### (c) Social association index

Using social association indexes, 131 pairs of species were identified with a mean ± s.e. association index of 0.17 ± 0.014. Indexes ranged from 0.03 to 0.9, with those closest to 1 indicating frequent co-occurrence within MPFAs (figure 3). The number of species co-occurring during MPFA events ranges from three to 19 with a mean of 11.9° ± 1.13 s.e. (supplementary material, table S2).

**Figure 3.**
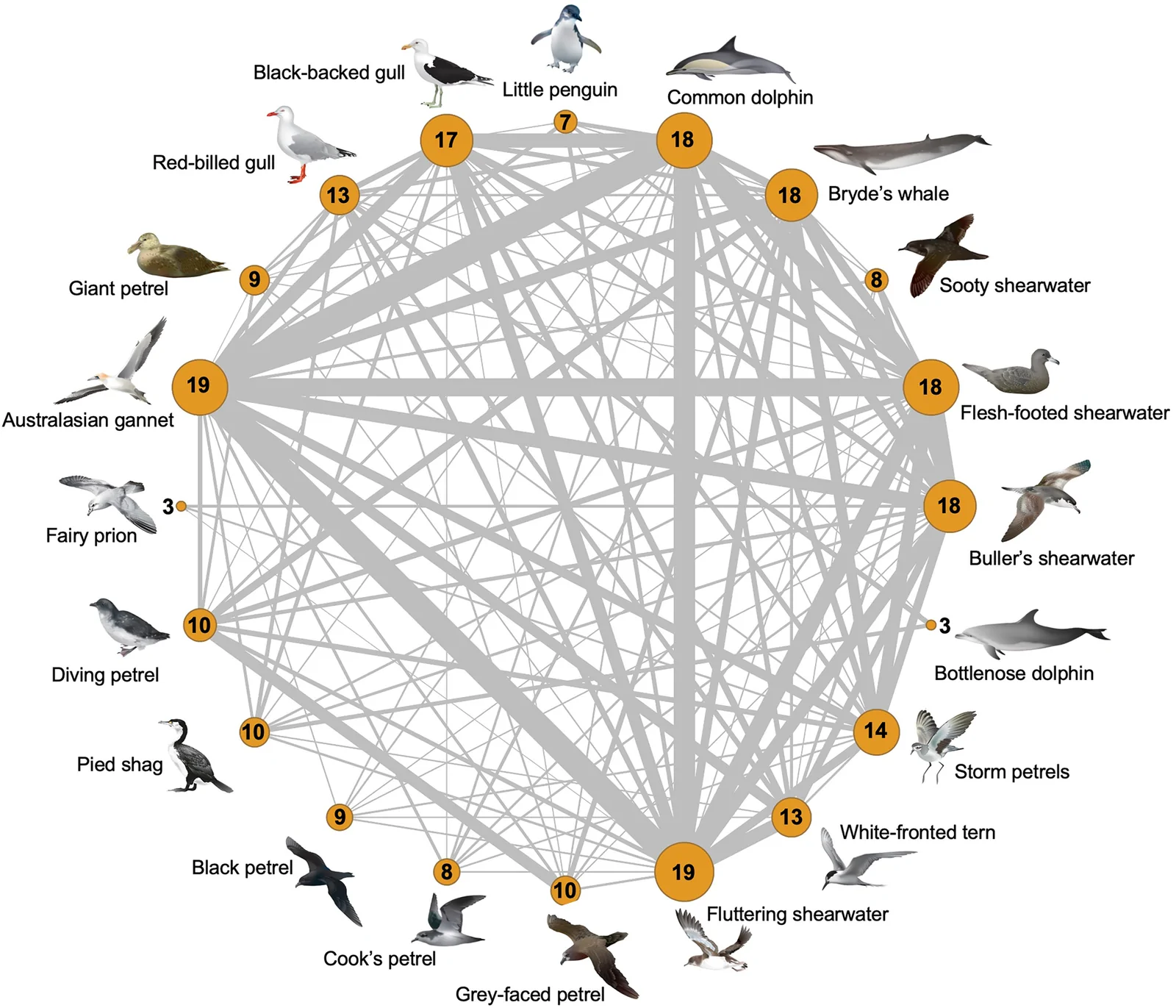
The social network of species associations within MPFAs (*n* = 179). Individual species’ circle (orange node) width includes weighted degrees (number of associates), while the grey linewidth is proportional to the association index between two species, with a wider line indicating a stronger association (supplementary material, table S2).

Association indexes varied between species (supplementary material, table S2) with the highest occurring between Australasian gannets and common dolphins (index: 0.9), gannets and fluttering shearwaters (index: 0.82) and fluttering shearwaters and common dolphins (index: 0.7). These high association rates reflect their frequent attendance within MPFAs (figure 3). High association indexes were also observed between Buller's and flesh-footed shearwaters (index: 0.61), flesh-footed shearwaters and common dolphins (index: 0.59) and flesh-footed shearwaters and gannets (index: 0.59).

### (d) Behavioural states

The proportion of time each species spends in the different behavioural states is highly variable between both species and clusters (supplementary material, figure S2). For each of the frequently attending (> 5% MPFA events) species, we found that cluster influenced (GLMM, ANOVA; *p* < 0.05) the proportion of time fluttering shearwaters, black-backed gulls and Buller's shearwaters spent engaging in all three behavioural states. No relationships between clusters and the proportion of time spent in each behavioural state (GLMM, ANOVA; *p* > 0.05) were found for Australasian gannets. Other species varied by behaviour and cluster (supplementary material, figure S2).

## 4. Discussion

Here, we reveal the complex dynamics within MPFAs in the Hauraki Gulf, New Zealand. The wide range of seabird and cetacean species that aggregate around ephemeral prey patches were dynamic and varied. As found in other ocean ecosystems, MPFAs vary in time and space. We found that association patterns changed during events, with the presence or absence of species affecting community dynamics. Some MPFA events were extensive, with large numbers of individuals shifting between patches within a feeding event. The size and dynamics of these events reflect less direct and obvious environmental changes, such as currents, upwellings and nutrient inputs, which were not included in this analysis but drive productivity in the Gulf [30,31]. Our demographic analysis focused on the presence and absence of species within MPFA events revealing a range of different community types and association patterns forming based on functional niches and foraging ecology. We use our behavioural observations and associations to interpret the species roles within MPFAs and how these changed with community composition and richness. Such roles vary; here, we simplify these to initiator/facilitator, maintainer, scavenger and disruptor/terminator.

### (a) Identifying communities in the Gulf

While it is challenging collecting accurate data on multiple species in a dynamic state, our research describes MPFAs in detail allowing consideration of species' roles and attendance. By incorporating fine-scale attendance patterns and species-level seabird identification, we expanded on previous broader findings [18] where data limitations meant seabird species were often aggregated by functional groups. We detected an increased number of communities (clusters) from three to eight, reflecting key associations. Using species-level identification when auditing communities allows for greater insight into the variety present, as when species are grouped by functionality, communities may be missed.

The eight communities (clusters) we identified were classified as planktivorous, scavengers or polytrophic using species-specific foraging ecologies and prey preferences. Polytrophic MPFAs (clusters d, e and h) were the most frequently encountered community type in the Gulf, with common dolphins and gannets probably facilitating foraging for various seabirds, including piscivores and planktivores via acoustic, visual and/or prey-herding behaviours, as shown in other studies [4,32,33]. Foraging niche partitioning allows similar species to forage within MPFAs, with their coexistence enabled through differences in foraging behaviours and preferred prey [15,34]. Foraging at different trophic levels within polytrophic MPFAs facilitates coexistence and minimizes competition between predators with similar foraging ecology [2,35]. Seabirds and dolphins typically associate with specific fishes, with feeding events in the Gulf involving many individuals foraging in the wake of or around schools, or travelling in a leap-frog formation, moving dynamically in response to changes to the fish school [36]. We commonly observed these polytrophic associations that predominantly included white-fronted terns and fluttering shearwaters, with shoaling fishes passively providing foraging cues for the terns and the terns providing cues for the shearwaters [36].

Anecdotal evidence suggests a substantial decrease in the frequency and size of MPFAs in the Gulf (R. Constantine 2026, personal observation), with the role of MPFAs in these animals' lives also changing. For example, the Bryde's whale shift in prey preference from primarily forage fish to zooplankton resulted in a substantial impact on the community structure in the Gulf [18]. MPFA dynamics reflect current ocean conditions and are a fragmented version of what MPFAs would have been before extensive anthropogenic pressures from fishing, habitat modification and land-based runoff caused substantial ecosystem disruption [37,38]. While our research reflects the new ‘normal’ Gulf, we expect these MPFA communities to change with further ecosystem disruption and climate-driven changes to ocean health [18,39].

### (b) Social associations: the role of species within MPFAs

Using demographic tools to determine species presence at MPFA events provides a holistic overview of the communities, enabling us to visualize and evaluate species’ connectivity and association within mixed-species groupings [29,40]. Species with similar foraging ecologies and dietary niches tend to more frequently forage together, with stronger social affinity indexes.

Dolphin–seabird associations were the most observed, with patterns probably reflecting foraging benefits for seabirds through prey herding behaviour of dolphins [1,6]. We found the strongest associations were between gannets, common dolphins and fluttering shearwaters. This was expected as the association of dolphins with gannets or other seabirds is well documented both within the Gulf [18,41] and elsewhere [1,3]. In the Gulf, the active herding behaviours of common dolphins counteracted the vertical scattering effect of aerial predators such as gannets that disturb the fish school with their diving behaviours. By contrast, the dolphins act as MPFA maintainers, cooperatively working to keep the fish prey aggregated and easily accessible [36]. Common dolphins were important facilitators of MPFA events, with shared prey herding probably reducing the energetic costs of foraging compared to solitary foraging. Subsurface predators play a unique role within MPFAs, with common dolphins, Bryde's whales and predatory fishes acting as facilitators, with distinct relationships forming between aerial and subsurface predators.

The role of species, however, is context dependent. For example, within Bryde's whale-storm petrel associations (cluster a) in the Gulf, the whales’ chin-slap foraging strategy aggregates zooplankton prey near the surface [42], with the whales acting as initiators/facilitators. This facilitation creates a unique opportunity for storm petrels which are not subsurface foragers [43] and are often foraging away from highly active MPFAs. By contrast, within polytrophic MPFAs (e.g. cluster h; supplementary material, figure S2), Bryde's whales were most frequently observed lunge feeding into the prey patch [18,42], halting MPFA activity by scattering and drastically reducing prey availability for other species, thus shifting the whales’ role to disruptors/terminators. The appearance of a disruptor or terminator can often bring foraging to an end, with the scattering or removal of prey reducing the benefits of foraging together [44,45].

These facilitator roles, however, were not just those who directly herd prey or change prey accessibility. We found associations also based on information transfer [6]. This relationship was highlighted in white-fronted tern and fluttering shearwater associations (clusters d and e). Here, the tern's zig-zag search patterns while flying and surface plucking indicated prey availability to the shearwaters which were typically sitting on the sea-surface and moved to the prey once the terns began feeding (R. Constantine 2019–2025, unpublished drone footage). The tern's behaviours serve as reliable cues for the fluttering shearwaters, which forage mainly during the day by sitting and dipping their head under the water as it is less energetically expensive than flying to find food [32,46]. Gannets also act as indicators of prey availability, with their high-flying and plunging behaviours attracting nearby predators, facilitating MPFA development [33].

Scavengers follow the MPFAs foraging on discards and prey made available by other predators. Their attendance is reflected in cluster b, which features black-backed (*Larus dominicanus*) and red-billed (*Chroicocephalus novaehollandiae scopulinus*) gulls. The role of these gulls in MPFAs is limited. They are not facilitators or maintainers, instead foraging alongside subsurface predators to obtain high-quality food through surface plucking on herded prey or scavenging on discards [47,48]. In some contexts, scavenging birds may have a scattering effect if kleptoparasiting on other foragers or acting as a perceived threat [45,49]. Northern giant petrels (*Macronectes halli*) and pied shags (*Phalacrocorax varius*) were occasionally observed foraging alongside other scavenging seabirds, foraging on prey scraps from messy piscivorous foragers, aligning with studies on other scavenging seabirds [50,51].

Larger fish also played a role in facilitating foraging opportunities for seabirds. These fish included trevally (*Pseudocaranx dentex*) and kahawai (*Arripis trutta*), which drive small prey to the surface [19,36]. Prey availability through associating with shoaling fish was probably a driver behind the strong foraging associations between fluttering shearwaters and white-fronted terns; however, we were unable to include these fishes in community analysis as they were difficult to accurately report from our research vessel, but drone imagery captured these associations for a future study.

### (c) Implications and conclusions

This study provides valuable insights into the fine-scale association patterns between seabirds and cetaceans in MPFAs, revealing eight unique MPFA communities in the Gulf. The identification of seabird associations at a species level and the combination of different analytical approaches expanded our understanding of MPFA drivers and dynamics, using traditional species-specific approaches applied to entire communities. The seabird–cetacean and seabird–seabird associations reflect mutualistic and commensal relationships, with feeding associations forming when targeting the same prey patches. Our study was limited to air-breathing ocean predators as predator–prey dynamics between those who spend most of their lives below the surface are challenging to observe from boat-based platforms. The use of drones to conduct continuous focal animal follows and automated machine learning tools can improve the detectability of underwater species and the accuracy of behavioural analyses [36]. In the Gulf, this would enable the inclusion of other subsurface predators such as sharks and rays [20] that are difficult to see from a boat. In the future, recording the duration of the observation period and the arrival/departure of species within MPFAs would help to further understand the formation of temporary communities and specific roles of each species.

Species have been associating and foraging within MPFAs long before human arrival. The role of these communities has shifted over time, with foraging dynamics acting as reliable indicators of cumulative ecosystem stressors and ocean health [10,41]. Variations in clusters and association indices reflect changing prey availability and oceanic conditions that influence prey movements and predators [17,19,20,52]. The study of MPFA dynamics and their changes in relation to oceanographic and environmental constraints, and anthropogenic stressors can facilitate a greater understanding of species roles and interconnectedness within communities offering a holistic overview of ecosystem health. Given the global similarity in community dynamics in pelagic MPFAs [1,2,21,53], our study has broader relevance beyond the Gulf which reflects a fragmented version of MPFAs before substantial pressure from human activities [37,38]. As megafauna reflect cumulative stressors in their environment, fine-scale drivers of MPFAs warrant further research. We suggest long-term monitoring of MPFAs, and a global meta-analysis would provide valuable insight into the effects of future climate and human-mediated changes on the resilience and dynamics of these important communities.

## Supplementary Material



## Data Availability

Data are available as supplementary material [54].

## Theme

One contribution of 14 to a theme issue ‘Multi-predator feeding aggregations in pelagic waters’.
