# Supplementary material for: Multi-predator feeding aggregations: understanding dynamic communities through functional niches and demographic analysis tools
Source: Philos Trans R Soc Lond B Biol Sci. 2026 Oct 15;381(1961):20250198. doi: 10.1098/rstb.2025.0198 (PMC13670079; doi:10.1098/rstb.2025.0198)
Supplement: Supplementary file 1 [file rstb.2025.0198.zip › rstb.2025.0198/rstb20250198_si_001.docx]

**Figure S1**. Multi-predator feeding aggregation (MPFA) attendance of clusters (a – h) by species (*n* = 22) with 100% being that the species attended all MPFA events categorised as that cluster.

Species codes are: ARDBUL – Buller’s shearwater; ARDCAR – flesh-footed shearwater; ARDGRI – sooty shearwater; BALBRY – Bryde’s whale; CHRNOV – red-billed gull; DELDEL – common dolphin; EUDMIN – little penguin; LARDOM – black-backed gull; MACHAL – giant petrel; MORSER Australasian gannet; PACTUR – fairy prion; PELURI – diving petrel; PHAVAR – pied shag; PROPAR – black petrel; PTECOO – Cook’s petrel; PTEGOU – grey-faced petrel; PUFGAV – fluttering shearwater; STESTR – white-fronted tern; STOPET – storm petrel; TURTRU – bottlenose dolphin; UNIDPE = unidentified petrel/shearwater; UNIDTE – unidentified tern
