# Supplementary material for: Multi-predator feeding aggregations: understanding dynamic communities through functional niches and demographic analysis tools
Source: Philos Trans R Soc Lond B Biol Sci. 2026 Oct 15;381(1961):20250198. doi: 10.1098/rstb.2025.0198 (PMC13670079; doi:10.1098/rstb.2025.0198)
Supplement: Supplementary file 1 [file rstb.2025.0198.zip › rstb.2025.0198/rstb20250198_si_002.docx]

**Table S1**. Overview of behaviours of cetaceans and seabirds within MPFAs.

| **State** | **Taxa** | **Group Behaviour** | **Description** |
| --- | --- | --- | --- |
| **Foraging** | Seabirds | High and low diving | Diving into the water from above the surface from various heights. Primarily used by piscivores. |
|  |  | Surface plucking /dipping | Picking prey from the water's surface by hovering above or dipping head under the water. Primarily used by planktivores. |
|  |  | Pursuit diving | Swimming under the water to chase prey. |
|  | Cetacean | Bubble-net | Release of bubbles while swimming to potentially entrap and aggregate prey. |
|  |  | Lunging | Specific for baleen whales. Rushing motion in the water to engulf prey, mouth extended to engulf water-laden prey. |
|  |  | Head/tail slaps | Breach and slap of head on the surface of the water. Tail flukes or head raised above the surface and ventral or lateral side slapped down. |
|  |  | Rushing/  Chasing | Rushing motion in the water to capture or engulf prey. |
| **Non-active non-foraging** | Seabird | Rafting | Sitting in a large single species group. |
|  |  | Sitting | Sitting on the surface of the water. |
|  | Cetacean | Milling | Directionless movement patterns, no net movement in any specific direction by individuals or the group. |
|  |  | Resting | Logging or slow, regular surfacing. Little evidence of forward movement, surfacing is more predictable, and exhalations are quiet |
| **Active non-foraging** | Seabird | Travelling | Flying in a single direction. |
|  |  | Approaching | Flying towards the MSFA. |
|  |  | Circling | Circling seabirds, frequently with head down while flying |
|  |  | Zig zagging | Flying as a coordinated group, often following subsurface prey |
|  |  | Chasing | Flying at/towards other seabirds, not associated with chasing prey |
|  | Cetaceans | Surfacing | Surfacing to breathe |
|  |  | Diving | Following breath on the surface, back is arched high, and animal disappears from view for a prolonged period (>1min). |
|  |  | Travelling | Engaging in persistent, directional movement, spacing is varied, and individuals swim with short, constant sub-surface intervals. |
|  |  | Socialising | Interactive events, tactile contact between individuals. Chasing, leaping, and/or engaging in physical contact. |
|  |  | Bow riding | Positioning themselves in the bow pressure wave of a moving vessel. |
|  |  | Fast travel | The entire body leaves the water, continuous jumping over >2 body lengths, fast travel |
|  |  | Mating | Belly to belly contact |
|  |  | Nursing | Calf positions itself under the mother and turns to suckle at the mammary slits. |
