# Supplementary material for: Multi-predator feeding aggregations: understanding dynamic communities through functional niches and demographic analysis tools
Source: Philos Trans R Soc Lond B Biol Sci. 2026 Oct 15;381(1961):20250198. doi: 10.1098/rstb.2025.0198 (PMC13670079; doi:10.1098/rstb.2025.0198)
Supplement: Supplementary file 1 [file rstb.2025.0198.zip › rstb.2025.0198/rstb20250198_si_003.docx]

**Table S2**. Association Indexes: Social affinity index between species (*n* = 22).

|  | **Buller’s shearwater** | **Flesh-footed shearwater** | **Sooty shearwater** | **Bryde’s whale** | **Red-billed gull** | **Common dolphin** | **Little penguin** | **Black-backed gull** | **Giant petrel** | **Australasian gannet** | **Fairy prion** | **Diving petrel** | **Pied shag** | **Black petrel** | **Cook’s petrel** | **Grey-faced petrel** | **Fluttering shearwater** | **White-fronted tern** | **Storm petrels** | **Bottlenose dolphin** |
| --- | --- | --- | --- | --- | --- | --- | --- | --- | --- | --- | --- | --- | --- | --- | --- | --- | --- | --- | --- | --- |
| **Flesh-footed shearwater** | 0.61 |  |  |  |  |  |  |  |  |  |  |  |  |  |  |  |  |  |  |  |
| **Sooty shearwater** | 0.18 | 0.12 |  |  |  |  |  |  |  |  |  |  |  |  |  |  |  |  |  |  |
| **Bryde’s whale** | 0.25 | 0.32 | 0.05 |  |  |  |  |  |  |  |  |  |  |  |  |  |  |  |  |  |
| **Red-billed gull** | 0.04 | 0.06 | 0 | 0.08 |  |  |  |  |  |  |  |  |  |  |  |  |  |  |  |  |
| **Common dolphin** | 0.33 | 0.59 | 0.05 | 0.25 | 0.14 |  |  |  |  |  |  |  |  |  |  |  |  |  |  |  |
| **Little penguin** | 0 | 0.11 | 0 | 0.05 | 0 | 0.14 |  |  |  |  |  |  |  |  |  |  |  |  |  |  |
| **Black-backed gull** | 0.22 | 0.24 | 0 | 0.16 | 0.19 | 0.47 | 0.04 |  |  |  |  |  |  |  |  |  |  |  |  |  |
| **Giant petrel** | 0 | 0.04 | 0 | 0.05 | 0.07 | 0.03 | 0 | 0.09 |  |  |  |  |  |  |  |  |  |  |  |  |
| **Australasian gannet** | 0.46 | 0.59 | 0.1 | 0.36 | 0.18 | 0.9 | 0.13 | 0.46 | 0.03 |  |  |  |  |  |  |  |  |  |  |  |
| **Fairy prion** | 0.1 | 0 | 0 | 0 | 0 | 0 | 0 | 0 | 0 | 0 |  |  |  |  |  |  |  |  |  |  |
| **Diving petrel** | 0.13 | 0.19 | 0 | 0.26 | 0 | 0.09 | 0 | 0.04 | 0.1 | 0.15 | 0 |  |  |  |  |  |  |  |  |  |
| **Pied shag** | 0.09 | 0.08 | 0 | 0 | 0.13 | 0.05 | 0 | 0.13 | 0 | 0.1 | 0 | 0 |  |  |  |  |  |  |  |  |
| **Black petrel** | 0.05 | 0.04 | 0 | 0.06 | 0 | 0.06 | 0 | 0 | 0 | 0.05 | 0 | 0 | 0 |  |  |  |  |  |  |  |
| **Cook’s petrel** | 0.08 | 0.08 | 0 | 0.11 | 0 | 0.08 | 0 | 0.04 | 0 | 0.1 | 0 | 0 | 0 | 0 |  |  |  |  |  |  |
| **Grey-faced petrel** | 0.04 | 0.08 | 0 | 0.17 | 0.07 | 0.06 | 0 | 0.04 | 0 | 0.1 | 0 | 0.38 | 0 | 0 | 0 |  |  |  |  |  |
| **Fluttering shearwater** | 0.41 | 0.54 | 0.05 | 0.27 | 0.19 | 0.7 | 0.14 | 0.41 | 0.05 | 0.82 | 0.03 | 0.14 | 0.08 | 0.06 | 0.06 | 0.08 |  |  |  |  |
| **White-fronted tern** | 0.26 | 0.11 | 0.04 | 0.1 | 0.17 | 0.17 | 0 | 0.23 | 0 | 0.35 | 0 | 0 | 0.05 | 0.05 | 0 | 0 | 0.45 |  |  |  |
| **Storm petrels** | 0.28 | 0.41 | 0 | 0.35 | 0 | 0.22 | 0.05 | 0.17 | 0.06 | 0.31 | 0.07 | 0.18 | 0 | 0.07 | 0.06 | 0 | 0.29 | 0.07 |  |  |
| **Bottlenose dolphin** | 0 | 0 | 0 | 0 | 0.17 | 0 | 0 | 0.04 | 0 | 0 | 0 | 0 | 0.14 | 0 | 0 | 0 | 0 | 0 | 0 |  |
