# Supplementary material for: Multi-predator feeding aggregations: understanding dynamic communities through functional niches and demographic analysis tools
Source: Philos Trans R Soc Lond B Biol Sci. 2026 Oct 15;381(1961):20250198. doi: 10.1098/rstb.2025.0198 (PMC13670079; doi:10.1098/rstb.2025.0198)
Supplement: Supplementary file 1 [file rstb.2025.0198.zip › rstb.2025.0198/rstb20250198_si_004.docx]

**Figure S2**.

For each of the frequently attending (>5% MPFA events) species, cluster influenced (GLMM, ANOVA; *p* < 0.05) the proportion of time fluttering shearwaters (PUFGAV), black-backed gulls (LARDOM), and Buller’s shearwaters (ARDBUL) spent engaging in all three behavioural states – active foraging, active non-foraging (e.g. flying) and non-active non-foraging (e.g. resting/sitting). No relationships between clusters and the proportion of time spent in each behavioural state (GLMM, ANOVA; *p* > 0.05) were found for Australasian gannets (MORSER). Other species varied by behaviour and cluster. Foraging and non-active non-foraging were found to vary by cluster for red-billed gulls (CHRNOV). For common dolphins (DELDEL), however, only foraging varied by cluster (*p* < 0.05). Non-active non-foraging was found to vary by cluster (*p* < 0.05) for flesh-footed shearwaters (ARDCAR), white-fronted terns (STESTR), and storm petrels (STOPET). And likewise, Bryde’s whales (BALBRY) showed significant cluster differences for active non-foraging (*p* = 0.03). No relationships between clusters and the proportion of time spent in each behavioural state (*p* > 0.05) were found for gannets.

**Figure S2**. The proportion of time frequently attending species (>5% multi-predator feeding aggregations (MPFAs); for attendance see table 1) spent foraging, active non-foraging (e.g. flying), non-active non-foraging (e.g. resting/sitting) and absent within MPFA events across the eight clusters (a - h). The middle of the box indicates median (50% quantile), with lower and upper hinges showing 25% and 75% quantiles, respectively. Data beyond the whiskers are “outliers” and plotted individually as dark grey points.

Species codes are: ARDBUL – Buller’s shearwater; ARDCAR – flesh-footed shearwater; BALBRY – Bryde’s whale; CHRNOV – red-billed gull; DELDEL – common dolphin; EUDMIN – little penguin; LARDOM – black-backed gull; MORSER Australasian gannet; PUFGAV – fluttering shearwater; STESTR – white-fronted tern; STOPET – storm petrel
